# Detection of Pandemic (H1N1) 2009 Virus in Patients Treated with Oseltamivir

**DOI:** 10.3201/eid1602.091328

**Published:** 2010-02

**Authors:** David Boutolleau, Nadhira Houhou, Claire Deback, Henri Agut, Françoise Brun-Vézinet

**Affiliations:** Université Pierre et Marie Curie 6, Paris, France; (D. Boutolleau, C. Deback, H. Agut); Pitié-Salpêtrière University Hospitals, Paris (D. Boutolleau, C. Deback, H. Agut); Bichat-Claude Bernard University Hospital, Paris (N. Houhou, F. Brun-Vézinet)

**Keywords:** Novel influenza A (H1N1) virus, RT-PCR, oseltamivir, influenza, viruses, expedited, France, letter

**To the Editor:** In April 2009, an influenza outbreak caused by a novel strain of influenza virus A (H1N1) was identified in Mexico. The rapid spread of this new virus among humans led the World Health Organization to raise the phase of pandemic alert to 6. We report results from the 2 virology laboratories from university hospitals that were involved in the surveillance network of pandemic (H1N1) 2009 in Paris at the beginning of the outbreak in France.

Patients exhibiting influenza-like illness (i.e., fever, sore throat, cough, asthenia, headache, myalgia) and who recently had traveled to countries where the pandemic (H1N1) 2009 outbreak had started (i.e., Mexico, United States, Canada, Japan) were hospitalized. Symptoms began either the day before or the day of hospitalization. Nasal-swab specimens were collected at admission by using the Virocult system (ELITech; Salon-de-Provence, France), and treatment with oseltamivir was started (75 mg, 2×/day). Pandemic (H1N1) 2009 infection was diagnosed by using rapid test QuickVue Influenza A+B (Quidel, San Diego, CA, USA) and real-time reverse transcription–PCR (RT-PCR) assays from the French National Influenza Centers or the US Centers for Disease Control and Prevention (*1*). In the case of a positive result, influenza virus in nasal secretions from patients was monitored daily by RT-PCR until viral genomes became undetectable.

From April 24 through June 7, 2009, nasal swab specimens from 234 persons (132 men; median age of all patients 33 years) were processed; pandemic (H1N1) 2009 infection was confirmed for 17 men and 15 women (median age 33 years) by RT-PCR. Results of the Quidel rapid tests were available for 27 specimens, with positive results for 9 (33% sensitivity). However, no positive result was observed with the Quidel rapid tests among the nasal-swab specimens with negative RT-PCR results (100% specificity). Influenza virus detection in nasal secretions was monitored for 16 patients who had laboratory-confirmed pandemic (H1N1) 2009 infection and were treated with oseltamivir. Viral detection by RT-PCR was absent 2 to >5 days after antiviral treatment began (Figure). Significant differences were not found in sex and age of the patients (data not shown).

**Figure:**
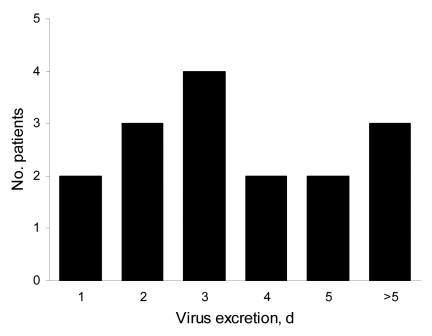
Duration of pandemic (H1N1) 2009 excretion in nasal swabs from patients treated with oseltamivir. The number of days from start of oseltamivir treatment to achievement of negative results of reverse transcription–PCR (RT-PCR) is indicated for 16 patients. The 3 patients classified in the last group (>5 days) are 1 patient with a negative RT-PCR result on day 7 posttreatment and 2 patients who still had positive results on day 5 posttreatment but provided no additional sample for testing.

These preliminary virologic data obtained during the first 6 weeks of pandemic (H1N1) 2009 in France confirm the poor sensitivity of the Quidel test toward this new virus, as recently reported (*2*). Further studies are needed to evaluate the performances of other rapid tests. Hayden et al. (*3*) demonstrated that treatment with oseltamivir significantly reduced duration of viral shedding among patients infected with seasonal influenza virus A (H1N1), in comparison with a placebo group: 1.5–2.5 days vs. 3.5–5.5 days (p = 0.003). In our study, surprisingly, PCR results for sequential nasal swab specimens from 16 patients infected by pandemic (H1N1) 2009 and treated with oseltamivir were negative within 3 days after therapy for only 9 (56%); indeed, for 3 (19%) patients, viral genome could be detected >5 days after antiviral treatment began. These data raise questions about potential virus transmission during antiviral treatment and the possible resistance of pandemic (H1N1) 2009 to oseltamivir. This latter point is now under study.
